# Anti-seizure gene therapy for focal cortical dysplasia

**DOI:** 10.1093/brain/awad387

**Published:** 2023-12-15

**Authors:** Amanda Almacellas Barbanoj, Robert T Graham, Benito Maffei, Jenna C Carpenter, Marco Leite, Justin Hoke, Felisia Hardjo, James Scott-Solache, Christos Chimonides, Stephanie Schorge, Dimitri M Kullmann, Vincent Magloire, Gabriele Lignani

**Affiliations:** Department of Clinical and Experimental Epilepsy, UCL Queen Square Institute of Neurology, University College London, London WC1N 3BG, UK; Department of Clinical and Experimental Epilepsy, UCL Queen Square Institute of Neurology, University College London, London WC1N 3BG, UK; Department of Clinical and Experimental Epilepsy, UCL Queen Square Institute of Neurology, University College London, London WC1N 3BG, UK; Department of Clinical and Experimental Epilepsy, UCL Queen Square Institute of Neurology, University College London, London WC1N 3BG, UK; Department of Clinical and Experimental Epilepsy, UCL Queen Square Institute of Neurology, University College London, London WC1N 3BG, UK; Department of Clinical and Experimental Epilepsy, UCL Queen Square Institute of Neurology, University College London, London WC1N 3BG, UK; Department of Clinical and Experimental Epilepsy, UCL Queen Square Institute of Neurology, University College London, London WC1N 3BG, UK; Department of Clinical and Experimental Epilepsy, UCL Queen Square Institute of Neurology, University College London, London WC1N 3BG, UK; Department of Clinical and Experimental Epilepsy, UCL Queen Square Institute of Neurology, University College London, London WC1N 3BG, UK; Department of Neuroscience, Physiology and Pharmacology, University College London, London WC1E 6BT, UK; Department of Clinical and Experimental Epilepsy, UCL Queen Square Institute of Neurology, University College London, London WC1N 3BG, UK; Department of Clinical and Experimental Epilepsy, UCL Queen Square Institute of Neurology, University College London, London WC1N 3BG, UK; Department of Clinical and Experimental Epilepsy, UCL Queen Square Institute of Neurology, University College London, London WC1N 3BG, UK

**Keywords:** epilepsy, focal cortical dysplasia, gene therapy, translation

## Abstract

Focal cortical dysplasias are a common subtype of malformation of cortical development, which frequently presents with a spectrum of cognitive and behavioural abnormalities as well as pharmacoresistant epilepsy. Focal cortical dysplasia type II is typically caused by somatic mutations resulting in mammalian target of rapamycin (mTOR) hyperactivity, and is the commonest pathology found in children undergoing epilepsy surgery. However, surgical resection does not always result in seizure freedom, and is often precluded by proximity to eloquent brain regions. Gene therapy is a promising potential alternative treatment and may be appropriate in cases that represent an unacceptable surgical risk. Here, we evaluated a gene therapy based on overexpression of the K_v_1.1 potassium channel in a mouse model of frontal lobe focal cortical dysplasia. An engineered potassium channel (EKC) transgene was placed under control of a human promoter that biases expression towards principal neurons (*CAMK2A*) and packaged in an adeno-associated viral vector (AAV9). We used an established focal cortical dysplasia model generated by *in utero* electroporation of frontal lobe neural progenitors with a constitutively active human Ras homolog enriched in brain (*RHEB*) plasmid, an activator of mTOR complex 1. We characterized the model by quantifying electrocorticographic and behavioural abnormalities, both in mice developing spontaneous generalized seizures and in mice only exhibiting interictal discharges. Injection of AAV9-*CAMK2A*-EKC in the dysplastic region resulted in a robust decrease (∼64%) in the frequency of seizures. Despite the robust anti-epileptic effect of the treatment, there was neither an improvement nor a worsening of performance in behavioural tests sensitive to frontal lobe function. AAV9-*CAMK2A*-EKC had no effect on interictal discharges or behaviour in mice without generalized seizures. AAV9-*CAMK2A*-EKC gene therapy is a promising therapy with translational potential to treat the epileptic phenotype of mTOR-related malformations of cortical development. Cognitive and behavioural co-morbidities may, however, resist an intervention aimed at reducing circuit excitability.

## Teaser

Focal cortical dysplasia (FCD) is the commonest pathology underlying malformations of cortical development resulting in intractable epilepsy. Almacellas Barbanoj *et al.* show that a gene therapy based on overexpression of the K_v_1.1 potassium channel is highly effective in reducing spontaneous seizures in a mouse model of FCD type II.

## Introduction

Focal cortical dysplasia (FCD) is a malformation of cortical development (MCD) and is among the leading causes of drug-resistant focal epilepsy pathology found in children undergoing epilepsy surgery.^1,2^ Because tissue is required to confirm the diagnosis, precise estimates of the prevalence are not available. Nevertheless, the European Epilepsy Brain Bank reported that 20% of specimens collected from therapeutic surgery corresponded to an MCD, of which 71% were classified as FCD.^1^

Surgical resection of the dysplastic focus is often indicated to treat patients with pharmacoresistant epilepsy associated with FCD. However, although surgery can be successful in 14–63% of operated cases,^3^ it is contraindicated in the majority of patients because of risks to normal brain function. There is therefore an urgent need for an effective therapy for FCD-associated epilepsy. The present study set out to test a gene therapy in a preclinical model.

FCD is classified histologically into several subtypes. FCD type II (FCD II) is characterized by dyslamination and dysmorphic neurons in a restricted area of the cortex and is generally caused by somatic mutations in neural progenitors.^4^ Approximately 60% of FCD II lesions are caused by mutations affecting the mTORC1 (mammalian target of rapamycin complex 1) signalling pathway.^2^ Despite advances in the molecular defects underlying FCD II, the epileptogenic mechanisms and the circuit disorders underlying cognitive and behavioural comorbidities that are frequently seen in affected children are incompletely understood.^2^

In recent years, new animal models of FCDs have been developed, which rely on activating the mTORC1 pathway through either downregulation of mTORC1 inhibitors (DEPDC5,^5^ PTEN,^6^ TSC1/2^7^) or upregulation of mTORC1 activators (Akt,^8^ Rheb,^9,10^ mTOR^11^). The tools used to modulate this pathway include CRISPR-Cas genome editing, cell-specific promoters, and *in utero* electroporation. These techniques allow for a focal, mosaic dysregulation of cortical development. In the present study, we used a mouse model of mTORC1-related FCD II generated by *in utero* electroporation of neuronal progenitors with a plasmid carrying a Ras homolog enriched in brain (*RHEB*) gene with a mutation (S16H) that increases mTORC1 activity.^9^ The *RHEB*^CA^ (constitutively active *RHEB*) plasmid was targeted to the frontal cortex unilaterally to mimic FCD II.

Overexpression of the voltage gated potassium channel K_v_1.1, encoded by *KCNA1*, has previously been shown to lead to a moderate decrease in neuronal excitability and neurotransmitter release from axonal terminals.^12^ Consequently, it has been validated as an effective gene therapy in several rodent models of focal epilepsy (focal neocortical epilepsy^13^ and temporal lobe epilepsy^14,15^), without off-target effects on a range of behaviours. Robust reductions in seizure frequency and/or duration were achieved by overexpressing wild-type *KCNA1* or an engineered version that bypasses the normal post-transcriptional editing of *KCNA1* mRNA and reduces inactivation (engineered potassium channel, EKC).^14^ An anti-epileptic effect has also been demonstrated by CRISPR-mediated upregulation of the endogenous murine *Kcna1* gene,^16^ and recently by using an activity-dependent promoter to drive EKC expression.^15^

Given the robust evidence for anti-seizure effects of exogenous EKC delivery in other models, we asked if it has a similar potential therapeutic effect in FCD II, and whether it affects cognitive and behavioural co-morbidities. We observed a substantial reduction in spontaneous seizures with no alteration in behavioural tests sensitive to frontal lobe function. EKC overexpression is thus a promising gene therapy to reduce seizure burden in FCDs. The absence of effect on behavioural tests supports the safety of the treatment, while underlining that co-morbidities may reflect a broader disorder of brain function in FCD.

## Materials and methods

### Animals

All procedures were performed in accordance with the UK Home Office Animals (Scientific Procedures) Act 1986. All mice were housed under a non-reversed 12 h:12 h light–dark cycle and had access to food and water *ad libitum*.

### *In utero* electroporation

The surgery was performed as previously described.^17^ Pregnant CD-1 dams [embryonic Day (E) 15 ± 0.5] (Charles River) were anaesthetized with isoflurane (induction 5%; maintenance 2.5%), and given Betamox® (15 mg/ml), buprenorphine (0.03 mg/ml) and Metacam® (0.15 mg/ml) intramuscularly.^17^ The dam was shaved, and an incision was performed close to the midline of the abdomen. The uterine horns were then exposed with a laparotomy. The CAG-tdTomato or CAG-tdTomato-T2A-*RHEB^CA^* plasmid (3.5 μg/μl) was mixed with the dye Fast Green (0.3 mg/ml, Sigma-Aldrich) and injected (∼1 μl/embryo) with a glass micropipette through the uterine wall unilaterally in the lateral ventricle of embryos. Then, three platinum electrodes soaked in sterile saline at ∼37°C were positioned around the embryo’s head [two tweezer-type circular electrodes (cathodes) on the temporal lobes and a third additional electrode (anode) on the frontal lobe]. The electroporation protocol consisted of one cycle of six electric pulses (28 V) of 100 ms duration with a 1 s inter-pulse interval (electroporator from Nepagene NEPA21 type II). The uterine horns were continuously moistened with warm sterile saline during the electroporation. After all the embryos were electroporated, the uterine horns were inserted back into the abdominal cavity. The dam was sutured and given 0.5 ml saline subcutaneously and placed in a recovery chamber for 30 min before being returned to its home cage. The animal was closely monitored for 10 days afterwards.

Between postnatal Days (P) 21 and 30, offspring were checked for tandem dimer Tomato (tdTomato) fluorescence with an epifluorescence stereomicroscope (Leica). The centre of the electroporated area was noted in reference to Bregma for further procedures and tdTomato-positive animals were single-housed. The overall success rate was 37%.

### Transmitter and cannula implantation

*RHEB* ^CA^ mice (weight 30–50 g) were anaesthetized with isoflurane (induction 5%, surgery 2.5%) and placed in a stereotaxic frame (Kopf). They were injected subcutaneously with buprenorphine (0.03 mg/ml) and Metacam (0.15 mg/ml). A wireless electrocorticogram (ECoG) transmitter (Cat. No. A3028C-CC single-channel transmitter, Open Source Instruments) was implanted subcutaneously with a subdural intracranial recording electrode positioned above the central point of the electroporated area. A reference electrode was implanted over the visual cortex in the contralateral hemisphere. A cannula (Plastics One) was positioned above the injection site for delivery of AAV vectors later during the experiment. The electrodes and cannula were then secured with dental cement. The animal was given a subcutaneous injection of 0.5 ml saline at the end of the surgical procedure and placed in a recovery chamber for 30 min before being returned to its home cage. The animal was then monitored daily for 5 days.

### Viral vector injection

After 2 weeks of baseline ECoG recording, mice were briefly anaesthetized with 2.5% isoflurane, and injected with 900 nl of adeno-associated virus serotype 9 (AAV9) expressing either *CAMK2A*-GFP or *CAMK2A*-EKC-GFP (with a titre of 4.81 × 10^12^ vg/ml), via the cannula.^14^ The injection was done at three depths to cover the entire cortex thickness (*z*: −1, −0.5, −0.3 mm) at 100 nl/min, with a 5 min pause between injections to avoid backflow. The animals were quarantined for 24 h before being returned to their home cage. The assignment of *CAMK2A*-GFP or *CAMK2A*-EKC-GFP to animals was randomized and the experimenter was blinded to the allocation.

### ECoG recording acquisition and analysis

ECoG was continuously recorded during a 15-day baseline, and then interrupted for 1 week during which behavioural tests were performed (see later), ending with viral vector injection. It was resumed 2 weeks later to allow for viral vector expression and then continued for a further 15 days. Data were acquired at a sampling rate of 256 Hz and band-pass filtered between 1 and 160 Hz. To reduce electrical noise and the risk of headstage dislocation, animals were individually housed in a Faraday enclosure.

Epileptiform activity was identified and classified automatically using custom-written MATLAB code. Frequency and time-domain features [variance, extreme values, coastline, and kurtosis and alpha/beta power (8–30), low gamma (20–40 Hz), high gamma (40–120 Hz), spectral entropy] extracted at two timescales (1 and 10 s) were used to calculate principal components, from which putative epileptiform activity was located and classified. Spikes not associated with continuous high-gamma band (40–100 Hz) activity were considered isolated ‘interictal spikes’, while groups of spikes associated with continuous high-gamma activity were considered ‘polyspikes’. Seizures were considered to be any activity which continued for at least 10 s, and which displayed a characteristic electrophysiological ‘morphology’ (Fig. 2D and Supplementary Fig. 2). Seizure morphology was defined as the presence of three stereotyped patterns in visually confirmed seizures in the *RHEB*^CA^ model: a sentinel spike, a rising or falling gamma band oscillation indicative of the tonic phase, and terminal rhythmic after-discharges in the low frequency range (1–5 Hz). ‘Generalized seizures’ were classified as activity which met the criteria for a seizure and showed increased signal amplitude with respect to baseline in the tonic phase. Spikes were identified and separated from probable electrical noise (fast, high amplitude deflections), using typical spectral properties. Briefly, short deviations from baseline were identified using a moving-window kurtosis measure, from which identified events, transient increase in theta power accompanied by sharp, transient increase in gamma power were found to reliably separate spikes from noise and putative seizures. In cases where spikes followed seizures, after-discharges of the seizure were considered to end when their frequency dropped below 1 Hz. These criteria reliably excluded intermittent periods of noise and electromyographic artefacts from analysis. Activity classified with a low confidence was manually confirmed (blind to experimental group).

To quantify the degree to which spikes were contaminated by physiological discharges (e.g. sharp wave rhythms), we applied the same criteria to recordings from control animals electroporated with tdTomato. The average frequency of spikes was 4.0 ± 2.0/h in tdTomato-electroporated mice versus 52.1 ± 24.7/h in tdTomato-*RHEB*^CA^ electroporated mice, implying a false discovery rate less than 8%.

In some animals ECoG acquisition was incomplete on some days for technical reasons. These days are indicated with an ‘X’ in the heat maps of Figs 2C and 5B and were not counted in calculating the cumulative seizure frequency plot in Fig. 5D.

### Video recordings

Cameras from Microseven (https://www.microseven.com/index.html) were used as previously described.^16^

### Behavioural assays

All behavioural tests were performed 1 h after the start of the light phase and 1 h before its end. When possible, the tests were performed under red light to reduce anxiety.

#### Spontaneous T-maze alternation

This test was performed as previously described.^18^ The apparatus made of transparent Plexiglass was cleaned with water after each animal. The walls of the apparatus were 20 cm high, with a full length and width of 50 cm. Each of the arms was 25 cm long, and the running track was 50 cm long. The protocol for T-maze alternation was adapted from the protocol previously described in Deacon and Rawlins.^18^ Mice were habituated to the room for 5–10 min before the experiment started. They were then placed at the starting area (bottom of the ‘T-shaped’ maze), facing away from the track. A central partition divided the end of the track to differentiate two directional choices. The animals initiated movement towards the apex of the T and chose one arm to enter. A positive entry was confirmed once the whole body (tail excluded) of the mice had entered the arm. The guillotine door was lowered to form an entrapped space for mice to stay for 30 s. Mice were then returned to the starting area and allowed to run down the track again with all doors open and without the separation wall in the middle. When the second arm was different from the first one chosen, it was counted as a positive alternation (score = 1), when it was the same it was counted as incorrect (score = 0). For each mouse, five successive trials were performed. The alternation rate was then calculated as:


(1) $$\frac{\text{Total score over trials 1-5}}{\text{5}}$$

If the animal had a behavioural seizure in the middle of the test, it was placed back in its home-cage, and returned to the arena after all the other animals had been tested.

#### Olfactory discrimination

The olfactory discrimination test was adapted from Yang and Crawley.^19^ The olfactory stimuli were prepared by diluting essential oils of peppermint, almond and raspberry in water (1:20). The solutions were freshly prepared and clean cotton swaps were briefly dipped in the different solutions. Both habituation and test were performed under white light. Mice were habituated for 30 min to an empty cage without bedding and a clean cotton swab inserted through the drinking bottle hole to remove any novelty confounder represented by the swab tip. The scented cotton swabs were then presented at the same height as the initial clean swab. Each odorant was presented three times, lasting 2 min for each trial, with an inter-trial interval of approximately 1 min (the time it took for the experimenter to change the cotton swab). The order of the scents was water, peppermint, almond (neutral smells) and two smells from the bedding of other mice of the same sex (social smells). To control for the test repetition after the therapy injection, the almond odour was substituted with raspberry scent and the social smells were from different mice. The animals were expected to show habituation to each repeated odorant across the three trials and to show a novelty response when faced with a different smell. In addition, they were expected to show more interest to the social smells than the neutral smells. The odour discrimination, habituation and interest were assessed by quantifying the time spent sniffing each cotton swab. The experimenter was blinded to the treatment groups.

### Immunofluorescence staining

#### Tissue collection

Mice underwent intracardiac perfusion with PBS followed by paraformaldehyde (PFA 4%). The brain tissue was kept overnight in PFA 4% after dissection, then washed with PBS before slicing. Coronal sections of 70 μm thickness from PFA-fixed mice brains were obtained using a vibrating microtome (Leica VT1000S) and stored in PBS with 0.02% sodium azide (NaN_3_) at 4°C.

#### Immunostaining and image acquisition

Free-floating brain sections were first washed three times for 5 min with PBS, followed by 3 h of incubation in blocking buffer (0.3% Triton X-100, 3% BSA and 5% goat serum). Slices were then incubated overnight at 4°C with a primary antibody in diluted blocking buffer (1 in 4) (1:1000, anti-rabbit pS6; #35708, Cell Signalling Technologies). Slices were then rinsed three times in PBS followed by secondary antibody incubation with Alexa Fluor 488 goat anti-rabbit (1:1000) diluted in PBS for 3 h at room temperature in the dark on a shaker. Slices were mounted and preserved using Vectashield antifade mounting medium with DAPI (Vector Laboratories). Tile-scan images were acquired with Zen software (Zeiss) on an LSM710 confocal microscope (Zeiss). Soma size was measured using FIJI software, and neuronal somata were outlined manually. Quantification of pS6 fluorescence intensity was performed using FIJI software, and the region of interest (ROI) was determined with a mask thresholding for the particles corresponding to neurons. For pS6 fluorescence quantification after treatment (Supplementary Fig. 2), electroporated neurons were excluded by applying a threshold to select cytomegalic neurons and by subtracting their grey values from the original image prior to pS6 raw intensity density quantification. The raw intensity density in the ROI was normalized to the number of particles.

The percentage of electroporated area (tdTomato) overlapping with *CAMK2A*-EKC transduced area (GFP+) was calculated applying the ‘Image Calculator’ tool in FIJI software between the red and green channels.

### Statistical analysis

Statistical analysis was performed on raw and normalized data with Prism (GraphPad Software). The statistical analysis performed is shown in each figure legend. Deviation from normal distributions was assessed using D’Agostino–Pearson’s test, and the F-test was used to compare variances between two sample groups. Student’s two-tailed *t*-test (parametric) or Wilcoxon test (non-parametric) was used as appropriate to compare means. One sample *t*-test was used to compare data to a specific value. To compare two groups at different time points, we used two-way repeated measure ANOVA or mixed-effects analysis, followed by Bonferroni *post hoc* test for functional analysis.

## Results

### The *RHEB*^CA^ mouse model recapitulates histological, electrophysiological and cognitive hallmarks of FCD II

**Figure 1:**
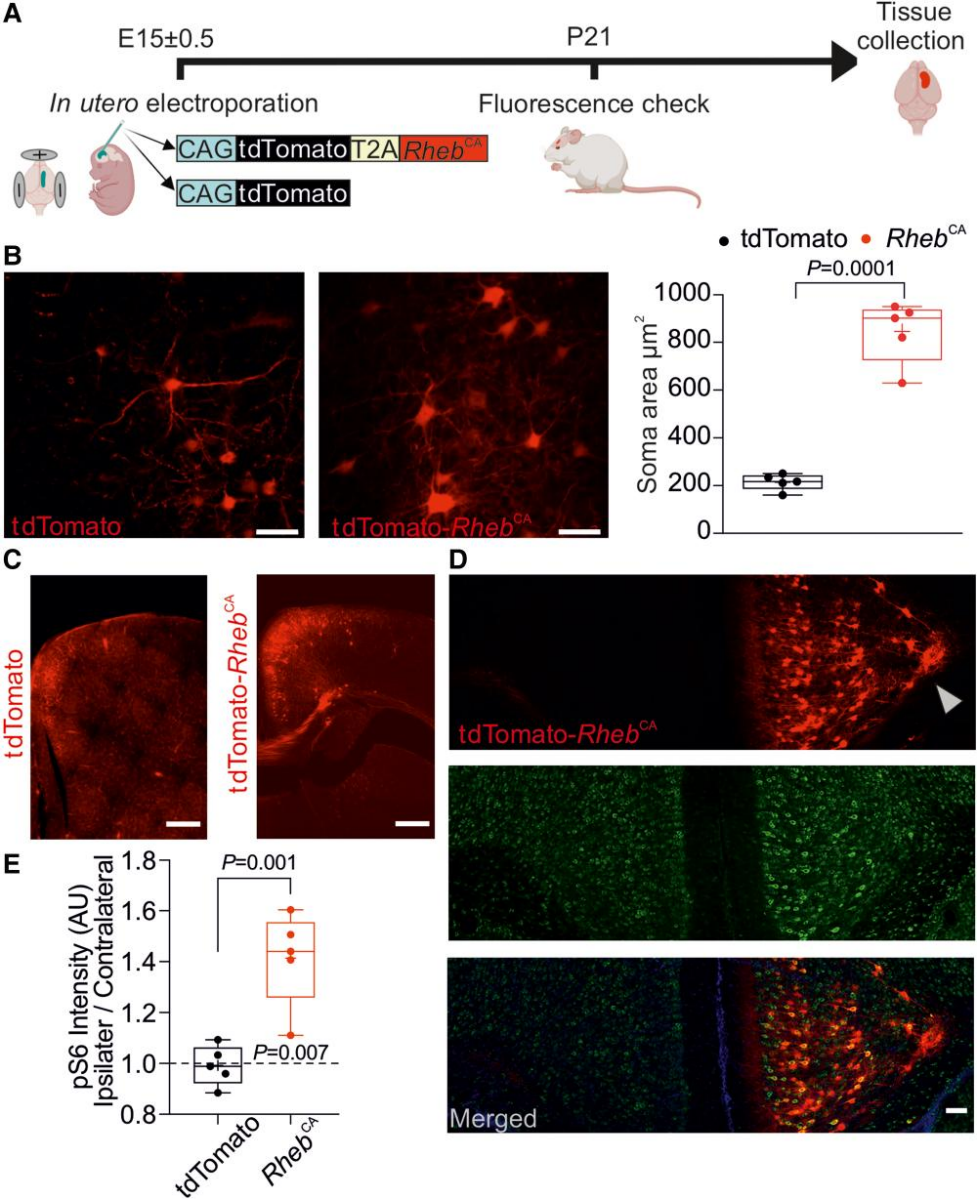
***RHEB* ^CA^ mouse model recapitulates histological hallmarks of focal cortical dysplasia type II.** (**A**) Experimental plan. E = embryonic Day; P = postnatal Day. Created with BioRender.com. (**B**) *Left and centre*: Fluorescence images of neurons expressing either tdTomato alone (control) or tdTomato-*RHEB*^CA^; scale bars = 50 μm. *Right*: Quantification of neuronal soma area (tdTomato, *n* = 5 mice, 2–5 slices per animal; *RHEB*^CA^, *n* = 5 mice, 2–4 slices per animal, unpaired Student’s *t*-test). (**C**) tdTomato or tdTomato-*RHEB*^CA^ prefrontal cortical slices; scale bars = 500 μm. (**D**) Representative coronal sections of prefrontal cortex in a *RHEB*^CA^ electroporated mouse. *Top*: tdTomato; *middle*: pS6; *bottom*: merged images. Note the dyslamination and heterotopic neurons in the *top* panel (arrowhead: heterotopic neurons in the corpus callosum); scale bars = 100 μm. (**E**) pS6 fluorescence intensity in tdTomato (control) or tdTomato-*RHEB*^CA^ with the ipsilateral (electroporated) normalized to the contralateral hemispheres (*n* = 5 mice, 2–4 slices per mouse, unpaired Student’s *t*-test and one-sample *t*-test versus 1). Data are plotted as box and whiskers, representing interquartile range (box), median (horizontal line), mean (+) and maximum and minimum (whiskers), together with all the points.

We first refined a previously reported model of FCD.^9^ We used a three-electrode *in utero* electroporation design^17^ with a plasmid expressing *RHEB*^CA^ together with a tdTomato fluorescent reporter linked by a cleavable T2A peptide (pCAG-tdTomato-T2A-*RHEB*^CA^). Mouse embryos were electroporated at E15 ± 0.5, and pups were screened with transcranial epifluorescence imaging at P21 to confirm unilateral tdTomato expression in the frontal lobe (Fig. 1A). As previously reported in this mouse model,^9^ we observed several characteristic features of FCD II in a subset of animals used for histology; electroporated neurons were heterotopic, dysmorphic and cytomegalic (Fig. 1B–E). Indeed, the average soma size of *RHEB*^CA^ cells was almost 3-fold greater than neurons electroporated with a control plasmid expressing only tdTomato (Fig. 1B). We also observed migration defects (Fig. 1C) and an increase in mTORC1 activity assessed by the quantification of S6 protein phosphorylation compared to the contralateral hemisphere (Fig. 1D and E and Supplementary Fig. 1).

**Figure 2:**
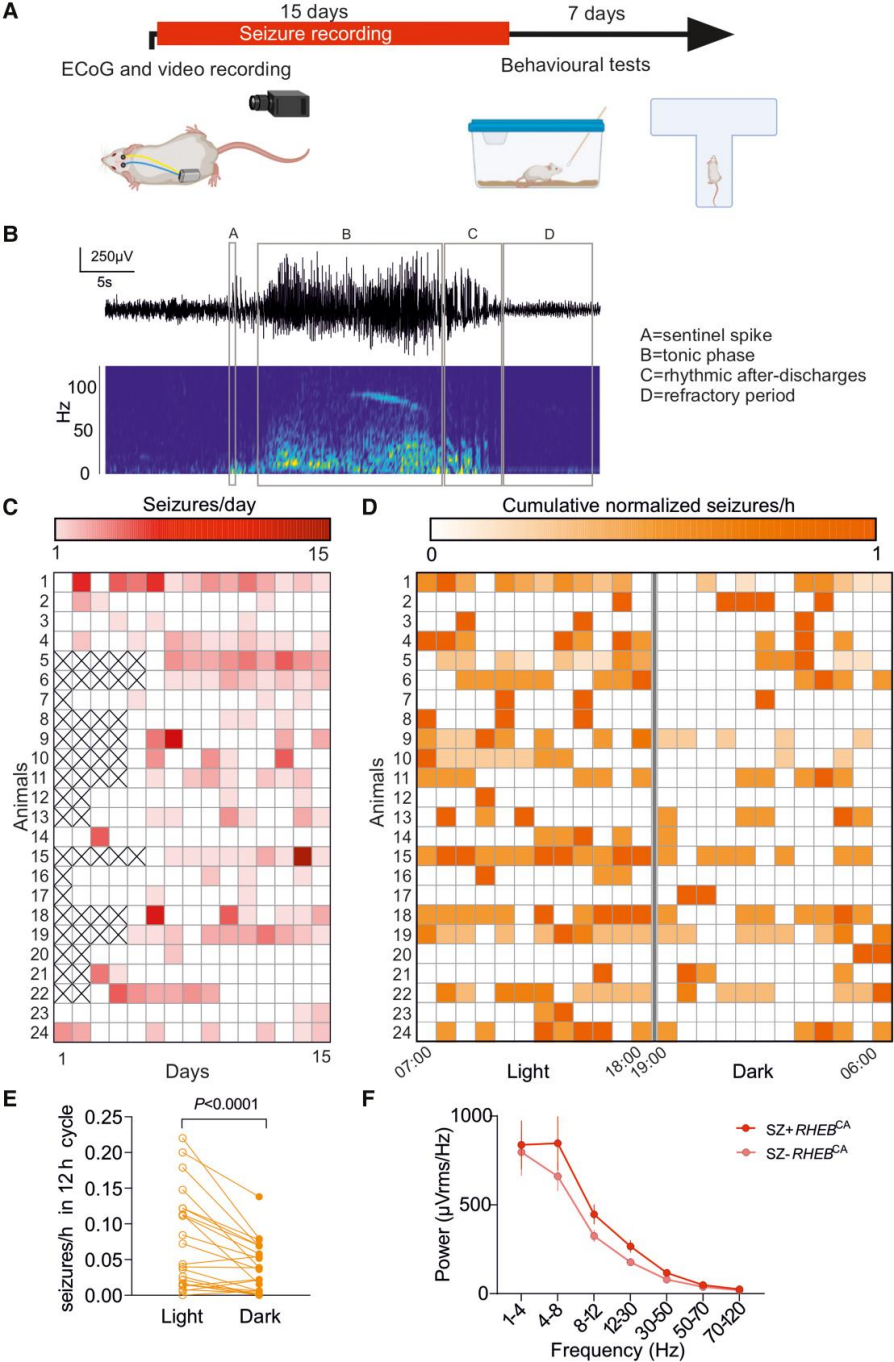
***RHEB* ^CA^ electroporated mice exhibit spontaneous generalized seizures preferentially during the light phase of the 24-h light-dark cycle**. (**A**) Timeline showing ECoG and video recording for quantification of seizures and behavioural tests (created with BioRender.com). (**B**) Representative seizure displaying the four main features used for automatic detection. (**C**) Raster plot showing the number for seizures for each animal (15 days, *n* = 24). (**D**) Raster plot of the distribution of the number of seizures per hour for each animal during the entire baseline recording period, normalized for each animal to the day with the maximum number of seizures (15 days, *n* = 24 animals). (**E**) Number of seizures normalized to recorded hours during the 12 h light and 12 h dark periods during 10–15 days shown for individual mice (*n* = 24, paired Wilcoxon test). (**F**) Power at different frequency bands in animals with (SZ+ *RHEB*^CA^  *n* = 24) and without (SZ− *RHEB*^CA^  *n* = 13) seizures.

We next characterized the epileptic phenotype of *RHEB*^CA^ electroporated animals, using subcutaneous wireless ECoG transmitters with a recording electrode over the tdTomato-positive area. After 10–15 days of baseline recording, (Fig. 2A) we observed several types of epileptiform activity that we classified according to their electrographic morphology (Supplementary Fig. 2; also see the ‘Materials and methods’ section). We only considered isolated epileptiform discharges not associated with detectable ongoing higher frequency activity as interictal spikes (Supplementary Fig. 2). Indeed, this type of event resembles repetitive epileptiform discharges seen in patients with FCD^20,21^ (Supplementary Fig. 2). In contrast, and unmistakable for interictal spiking or polyspikes,^21^ seizures are associated with a pronounced sentinel spike followed by a high frequency ‘chirp’ in the gamma band which resembles the typical low voltage fast activity (LVFA) onset pattern observed in the majority of patients suffering from FCD^21,22^ (Fig. 2B and Supplementary Fig. 2). This first period, corresponding to the tonic phase of the seizure, gives way invariably to high-amplitude spike-and-wave discharges at a decreasing frequency (down to 2–4 Hz), until seizure termination (Fig. 2B and Supplementary Fig. 2).

A subset of animals underwent concurrent video monitoring, which showed that seizures detected using our custom algorithm corresponded to generalized tonic-clonic seizures with convulsions (Racine scale 5). These generalized seizures started with behavioural arrest, followed by either fore-limb jerking or stereotypical rotation before generalized tonic-clonic activity with loss of postural tone (Supplementary Video 1). In the present study, we focused only on spikes and generalized seizures because of their clinical relevance and unambiguous electrographic morphology.

Overall, we observed generalized seizures in 24 of 37 *RHEB^CA^*-electroporated animals (∼65%). Epileptic animals had a median seizure frequency of 0.9 per day (Fig. 2B and C), less than previously reported using a similar experimental design.^23^ Among possible explanations for the lower seizure frequency is the introduction of the T2A linker in the plasmid construct.^24^ Epileptic animals exhibited ∼57% seizure-free days during a 15-day recording period (Fig. 2C). Seizure occurrence was greatest during the light phase of the 12 h:12 h light-dark cycle (Fig. 2D and E). Given that mice tend to be more active during the dark phase, this finding is reminiscent of the propensity for nocturnal seizures in patients with frontal lobe epilepsy.^25^ We also observed a non-significant trend towards an increase in ECoG power in animals with seizures compared to those without (SZ+ *RHEB*^CA^ and SZ− *RHEB*^CA^, respectively) (Fig. 2F).

**Figure 3:**
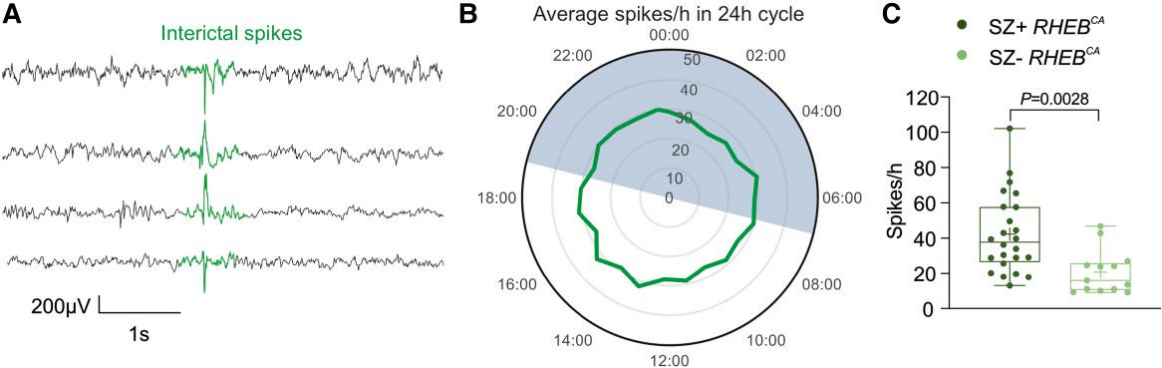
**The *RHEB*^CA^ mouse model displays features of cortical hyperexcitability.** (**A**) Representative traces from four different animals showing interictal spikes (aligned at centre). (**B**) Clock graph displaying the average number of spikes per hour recorded during the 12 h:12 h light-dark cycle; mice with and without generalized seizures (SZ+ *RHEB*^CA^ and SZ− *RHEB^CA^*, respectively) were included in this analysis (*n* = 38 mice). The dark phase was between 19:00 and 07:00. (**C**) Box plot showing the number of interictal spikes per hour (SZ− *Rheb*^CA^  *n* = 13; SZ+ *Rheb*^CA^  *n* = 24; unpaired Student’s *t*-test). Data are plotted as box and whiskers, representing interquartile range (box), median (horizontal line), mean (+) and maximum and minimum (whiskers), together with all the points.

We also examined the occurrence of spikes (Fig. 3A), which are a frequent biomarker of focal epilepsy,^26^ in *RHEB*^CA^ electroporated animals either with (SZ+ *RHEB*^CA^) or without (SZ− *RHEB*^CA^) generalized seizures. In contrast to seizures, interictal spikes displayed no obvious relationship to the light-dark cycle (Fig. 3B). However, SZ+ *RHEB*^CA^ animals had a higher frequency of spikes than SZ− *RHEB*^CA^ mice (Fig. 3C).

**Figure 4:**
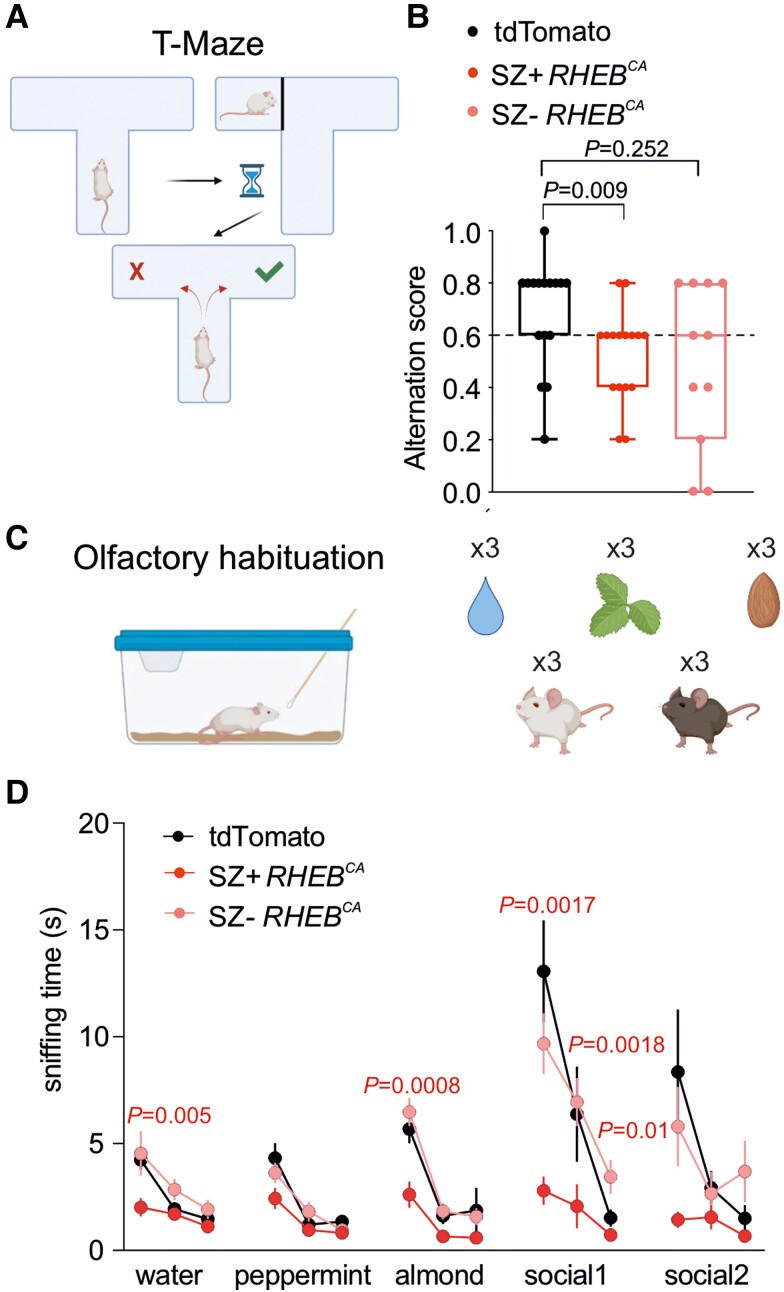
***RHEB* ^CA^ mice have cognitive impairments.** (**A**) T-maze test (created with BioRender.com). (**B**) Box plot displaying the average alternation score in the T-maze test for td-Tomato (*n* = 16), SZ+ *RHEB^CA^* (*n* = 16) and SZ− *RHEB*^CA^ (*n* = 11) mice [Fisher’s exact test (>0.6 versus ≤0.6), tdTomato versus SZ+ *RHEB*^CA^ or SZ− *RHEB*^CA^]. Data are plotted as box and whiskers, representing interquartile range (box), median (horizontal line) and maximum and minimum (whiskers), together with all the points. (**C**) Olfactory habituation test. Each smell [water, peppermint, almond and other mice (social)] was presented three times (created with BioRender.com). (**D**) Graph displaying the sniffing time of three neutral smells (water, peppermint, almond) and two social smells in tdTomato (*n* = 16), SZ+ *RHEB^CA^* (*n* = 17) and SZ− *RHEB^CA^* (*n* = 11) mice. Repeated measures two-way ANOVA (Experimental group × Trial factor *P* < 0.0001; trial factor *P* < 0.0001; experimental group factor *P* = 0.0005) followed by Bonferroni multiple comparison test.

Behavioural and cognitive co-morbidities including learning disability and high autism spectrum quotient are common in FCD patients.^27,28^ We therefore examined spontaneous alternation in a T-maze (Fig. 4A and B) and performed tests of olfactory discrimination (Fig. 4C and D), both of which are reliant on prefrontal cortex function,^29,30^ in SZ− *RHEB^CA^* and SZ+ *RHEB*^CA^ animals. Interestingly, SZ+ *RHEB*^CA^ animals exhibited a significant impairment in alternation in the T-maze and less time spent investigating neutral odours and social olfactory cues compared to tdTomato animals (Fig. 4B and D), consistent with learning disability and impaired social cognition often seen in patients with prefrontal FCD.^27,28^

### *CAMK2A-*EKC gene therapy reduces seizures without affecting cognition

**Figure 5:**
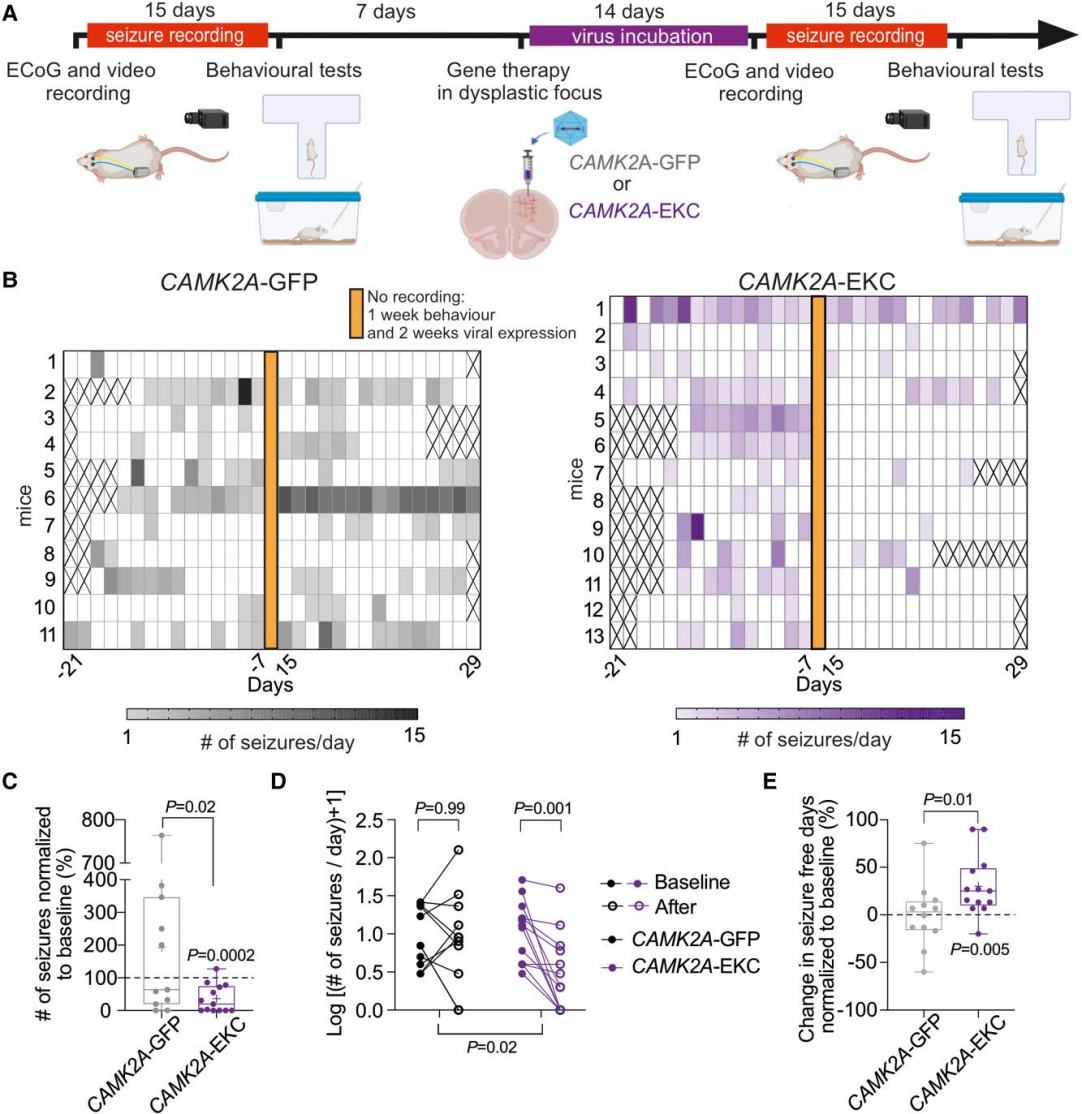
***CAMK2A*-EKC therapy reduces seizure frequency.** (**A**) Timeline (created with BioRender.com). (**B**) Heat map displaying seizure occurrence in each animal injected with either *CAMK2A*-GFP (grey) or *CAMK2A*-EKC virus (purple) before and after the treatment (virus injection on Day 0). Crosses correspond to days where data acquisition was incomplete. (**C**) Number of seizures normalized to baseline for either animals treated with *CAMK2A*-GFP or *CAMK2A*-EKC (unpaired Student’s *t*-test and one-sample *t*-test versus 100%). Data are plotted as box and whiskers, representing interquartile range (box), median (horizontal line), mean (+) and maximum and minimum (whiskers), together with all the points. (**D**) Slope graph displaying the change in seizure frequency for animals injected with *CAMK2A*-EKC (*n* = 13 mice) or *CAMK2A*-GFP (*n* = 11 mice) displayed as log(seizures + 1). Two-way ANOVA followed by Bonferroni multiple comparison test. (**E**) Graph showing the percentage of seizure-free days normalized to baseline (unpaired Student’s *t*-test and one-sample *t*-test versus 0).

The *RHEB*^CA^ model thus has both construct and face validity for FCD II. We asked whether expressing EKC in the dysplastic region to decrease neuronal and circuit excitability attenuates seizures. After recording 15 days of baseline ECoG (Fig. 2), we injected either AAV9-*CAMK2A*-EKC-GFP or a control virus expressing GFP alone, AAV9-*CAMK2A*-GFP, in the electroporated frontal lobe for a biased expression in excitatory neurons^31^ (Fig. 5A). Animals (from Fig. 2C) were randomized to the treatment groups and the experimenter was blinded to the allocation. ECoG recording was suspended for 2 weeks to preserve transmitter battery life and to allow for transgene expression before resuming for another 15 days. Both SZ+ *RHEB^CA^* and SZ− *RHEB^CA^* animals were injected. In a subset of animals, we analysed the spatial expression of the reporter protein as well as pS6 expression. Overall, we observed expression of the viral reporter GFP in 22 ± 7% (*n* = 5) of the tdTomato-positive area, but also outside the dysplastic region, with considerable inter-animal differences (Supplementary Fig. 3A).

We observed no difference in pS6 expression between the *CAMK2A*-EKC and *CAMK2A*-GFP groups (Supplementary Fig. 3B).

Epileptic *RHEB^CA^* animals (SZ+ *RHEB*^CA^) injected with AAV9-*CAMK2A*-EKC-GFP showed a robust decrease in seizures in relation to their baseline (−64% ± 12%, *n* = 13 mice, *P* = 0.0002, one-sample paired *t*-test; Fig. 5C and D and Supplementary Fig. 4). In contrast, animals treated with the control virus AAV9-*CAMK2A*-GFP showed an increase in seizures, although this fell short of significance (+192% ± 71%, *n* = 11 mice, *P* = 0.22, one sample paired *t*-test; Fig. 5B–D). When this spontaneous evolution of seizure frequency was taken into consideration, we estimated that *CAMK2A*-EKC decreased seizures by ∼87% (Supplementary Fig. 4). Overall, 60% of EKC-treated SZ+ *RHEB*^CA^ mice became seizure-free by the last week of recording (Fig. 5B), and there was a 31% increase in seizure-free days (Fig. 5E). The effect of the treatment was also evident when plotting the cumulative evolution of seizures after treatment, normalized by each animal’s baseline (Supplementary Fig. 4). No effect on seizure duration was however seen (Supplementary Fig. 5). The light-dark cycle pattern of seizure occurrence was maintained in *CAMK2A*-EKC treated animals although was not evident in *CAMK2A*-GFP-treated animals (Supplementary Fig. 6).

**Figure 6:**
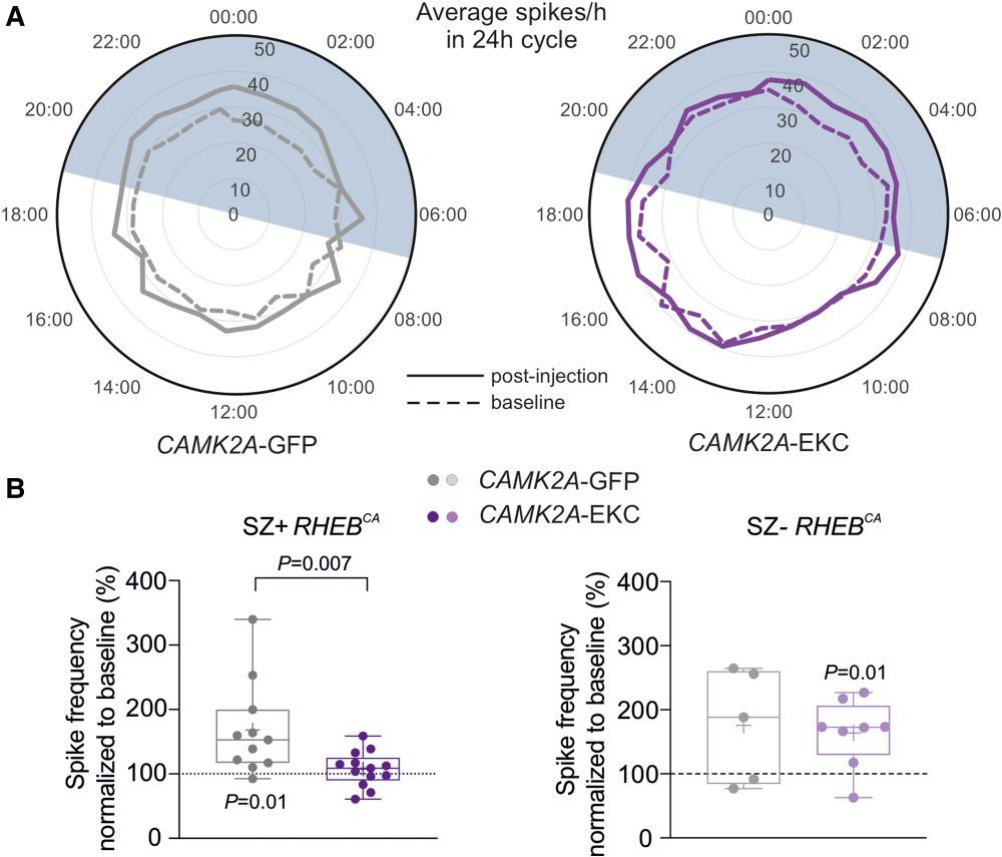
**Effects of *CAMK2A*-EKC therapy on spikes in *RHEB^CA^* animals with and without seizures.** (**A**) Circular graph displaying the average number of spikes per hour before (dashed line) and after the therapy (continuous line) over 24 h cycles. (**B**) Box plot displaying average spike frequencies, normalized to baseline, in SZ+ *RHEB*^CA^ animals (*CAMK2A*-GFP *n* = 11 mice, *CAMK2A*-EKC *n* = 13 mice; unpaired Student’s *t*-test and one-sample *t*-test versus 100%) and in SZ− *RHEB*^CA^ animals (*CAMK2A*-GFP *n* = 5 mice, *CAMK2A*-EKC, *n* = 8 mice). Data are plotted as box and whiskers, representing interquartile range (box), median (horizontal line), mean (+) and maximum and minimum (whiskers), together with all the points.

We also assessed the effect of the treatment on spikes. These continued to show no obvious diurnal variability in either treatment groups (Fig. 6A). There was, however, a relative decrease in the frequency of spikes after treatment with *CAMK2A*-EKC in SZ+ *RHEB^CA^* mice when compared to CAMK2A-GFP treated mice (*CAMK2A*-GFP: 168.1 ± 21.9%, *n* = 11; versus *CAMK2A*-EKC: 107.4 ± 7.5%, *n* = 13), while SZ− *RHEB^CA^* animals showed no difference (*CAMK2A*-GFP: 76.9 ± 39.6%, *n* = 5; versus *CAMK2A*-EKC: 62.7 ± 18.6%, *n* = 8) (Fig. 6B and Supplementary Fig. 7).

**Figure 7:**
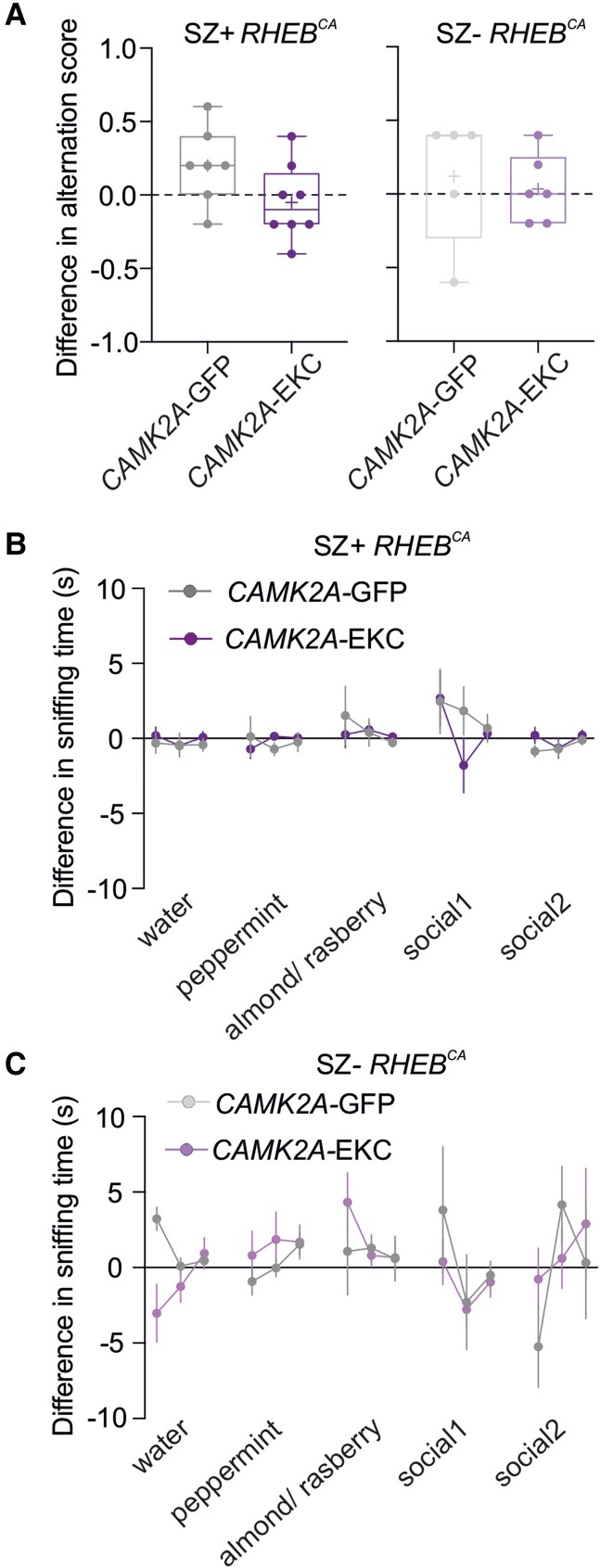
***CAMK2A*-EKC therapy neither aggravates nor worsens behavioural deficits in the *RHEB*^CA^ model.** (**A**) Box plot showing the difference in alternation scores in the T-maze of *CAMK2A*-GFP (*n* = 7) or *CAMK2A*-EKC (*n* = 8) treatment in SZ+ *RHEB*^CA^, and *CAMK2A*-GFP (*n* = 5) or *CAMK2A*-EKC (*n* = 6) treatment in SZ− *RHEB*^CA^, compared to baseline. Each circle represents an individual animal (one-sample *t*-test versus 0 and two-way ANOVA, *P* = 0.1407). Data are plotted as box and whiskers, representing interquartile range (box), median (horizontal line), mean (+) and maximum and minimum (whiskers), together with all the points. (**B**) Sniffing time for three neutral smells and two social smells before and after either *CAMK2A*-GFP (*n* = 7) or *CAMK2A*-EKC (*n* = 8) treatment in SZ+ *RHEB*^CA^ [one-sample *t*-test versus 0 (α = 0.003, corrected for multiple comparison) and repeated measurement mixed-effects analysis, *P* = 0.37]. (**C**) Sniffing time for three neutral smells and two social smells before and after either *CAMK2A*-GFP (*n* = 5) or *CAMK2A*-EKC (*n* = 6) treatment in SZ− *RHEB^CA^* [one-sample *t*-test versus 0 (α = 0.003, corrected for multiple comparison) and repeated measurement mixed-effects analysis, *P* = 0.98].

Finally, we asked whether the gene therapy affects the behavioural phenotype observed before treatment (Fig. 4). Neither the working memory test nor the social discrimination task was altered after treatment with *CAMK2A*-EKC or *CAMK2A*-GFP in animals with seizures (Fig. 7A and B). Similarly, no differences in behaviour were seen in animals without seizures after treatment with *CAMK2A*-EKC or *CAMK2A*-GFP (Fig. 7A and C).

## Discussion

The main finding of the present study is that gene therapy with *CAMK2A*-EKC is highly effective in reducing spontaneous generalized seizures in the *RHEB*^CA^ model of FCD II, while neither worsening nor improving cognitive and behavioural comorbidities.

The study underlines the construct, face and predictive validity of the *RHEB*^CA^ model (Figs 1–3).^9,32^ Dysregulation of the mTORC1 pathway leads to defects in migration of neural progenitors and cellular and circuit abnormalities.^2^ We extend the characterization of the *RHEB*^CA^ mouse model by showing that it has significant impairments in working memory and social recognition (Fig. 3). Although cognitive comorbidities accompany several forms of epilepsy and have been attributed to some extent to the acute effects of seizures and anti-seizure medication, they are especially common in ‘mTORopathies’ including FCD. In this study we aimed to reproduce as closely as possible a clinical scenario where treatment would be given following the emergence of seizures. The absence of significant changes in both behavioural tests is encouraging with respect to the safety of our therapy. On the other hand, failure to improve these aspects of the phenotype argues that they arise from the underlying circuit dysfunction rather than from seizures *per se*. Importantly, we showed that animals with spontaneous generalized seizures present severe cognitive defects (Fig. 4), suggesting that although these impairments could be associated with the structural malformations and/or mTOR hyperactivity, seizures during development are a key factor for the manifestation of the behavioural phenotype. It remains to be determined whether an intervention earlier in life could prevent circuit dysfunction and thus mitigate behavioural deficits.

Although EKC treatment was highly effective, there was a relatively low spatial overlap between dysplastic neurons and virus expression. This may have reflected difficulty targeting the dysplastic zone reliably when guided only by tdTomato fluorescence. However, alternative possible explanations include limited tropism of the AAV9 capsid for dysplastic neurons, and/or limited ability of the *CAMK2A* promoter to drive expression of transgenes. Differences in AAV tropism towards neurons in mouse models have been previously reported^33^ and neurons carrying the *RHEB* (S16H) mutation have altered membrane properties.^34^ Importantly, this low overlap, in combination with the decrease of the number of generalized seizures observed in the *CAMK2A*-EKC group, suggests that treating the periphery of the FCD area is sufficient to prevent generalisation of seizures. However, we also found that the EKC treatment was more effective at preventing an increase in the spike frequency from baseline in animals exhibiting seizures, than in animals without seizures. The effect of EKC therapy would therefore be consistent with a reduction in network excitability at the focus since spikes are thought to be generated locally.^35^

As mentioned above, anti-seizure treatment using EKC (or upregulating endogenous *Kcna1* transcription) has been previously reported in models of temporal lobe epilepsy.^15,16^ The present study adds FCD II to the range of epilepsy models in which gene therapy with *CAMK2A*-EKC is effective. Although overactivation of the mTOR pathway is the commonest signalling disorder underlying FCD II, it is not the only one, and in clinical practice establishing the ultimate cause is only rarely possible without sampling brain tissue from the lesion. Nevertheless, the success of the *CAMK2A*-EKC reported here supports the potential use of the treatment in focal epilepsy irrespective of its aetiology. Promising results of ion channel-based gene therapy have previously been reported in the same model of FCD II: a treatment aimed to counteract overexpression of the hyperpolarization-activated cyclic nucleotide–gated potassium channel isoform 4 (HCN4) was effective in reducing seizure occurrence.^34^ Nevertheless, it remains to be determined whether this treatment’s effectiveness is limited to epilepsies caused by overactivation of mTORC1.

Interestingly, an increase in mTORC1 activity has been linked with alterations in K_v_1.1 expression.^36-39^ The interpretation of these studies is complex, as they suggest both an mTORC1-dependent and an independent regulation of K_v_1.1 secondary to seizures, underlining the multifaceted nature of the mTOR pathway.^40^ Indeed, an increase or a decrease in K_v_1.1 hippocampal expression has been observed depending on the model used, and more work is needed to understand whether K_v_1.1 expression is altered in FCD II associated with mTOR overactivation.^36,38^

Although invasive, injection of an AAV vector is likely to be a more palatable option than surgical resection. Indeed, the focal nature of FCD, which can frequently be detected on MRI, provides a target for AAV delivery. The local transduction of neurons avoids one of the main limitations of drug treatment, whether using anti-seizure medication or mTOR-inhibiting drugs such as sirolimus (rapamycin) or its analogue everolimus, namely the risk of side effects arising from actions throughout the body.^41^ In addition, whilst rapamycin and its analogues need to be taken continuously, gene therapy aims for a long-term solution in one intervention.

In conclusion, overexpression of the engineered potassium channel is an effective approach to treat seizures in FCD II. We provide a robust and safe treatment option, potentially suitable for clinical translation, for one for the commonest causes of refractory epilepsy.

## Supplementary Material

awad387_Supplementary_Data

## Data Availability

Data and materials availability: All Python scripts will be made available online, and all the vectors used in this manuscript will be made available on a suitable platform with a material transfer agreement. All raw data supporting the main and supplementary figures are available upon request.
