# Supplementary material for: Anti-seizure gene therapy for focal cortical dysplasia
Source: Brain. 2023 Dec 15;147(2):542–53. doi: 10.1093/brain/awad387 (PMC10834237; doi:10.1093/brain/awad387)
Supplement: awad387_Supplementary_Data [file awad387_supplementary_data.pdf]

Supplementary figures

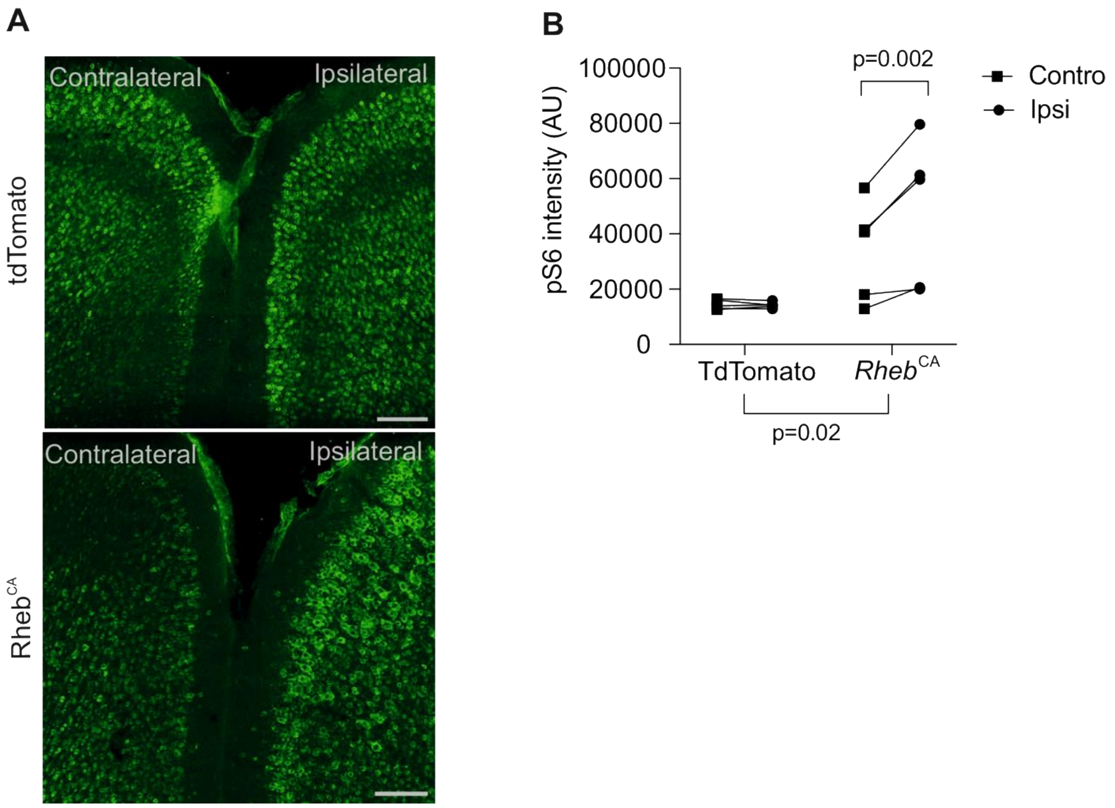

Fig.S1. *Rheb*^{CA} mice shows higher pS6 fluorescence intensity in the electroporated (ipsilateral) hemisphere compared to tdTomato (control). A. Representative images of pS6 fluorescence in tdTomato or tdTomato-*RHEB*^{CA} mice. **B.** Slope graph of pS6 intensity for tdTomato or tdTomato-*RHEB*^{CA} mice (n=5 mice, 2-4 slices per mouse, 2-way ANOVA followed by Bonferroni multiple comparison test).

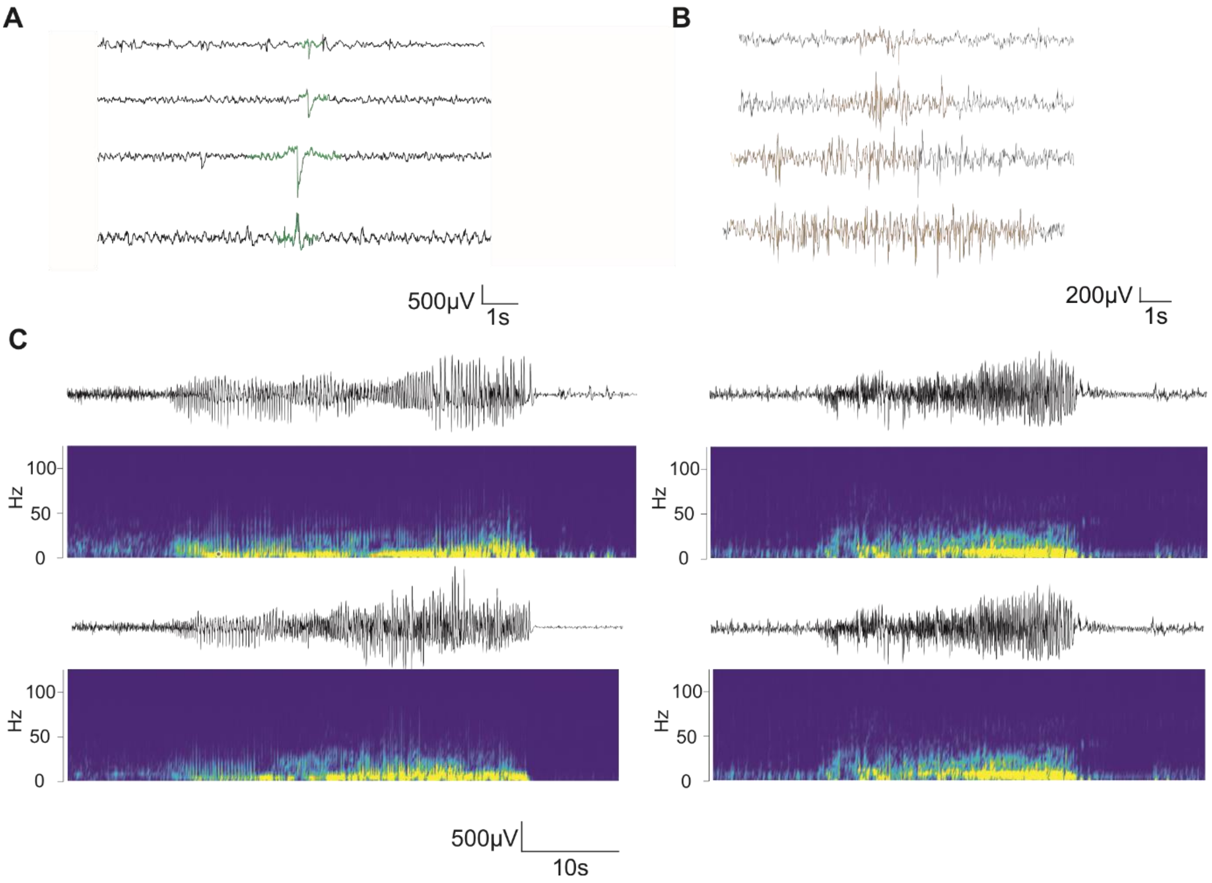

Fig.S2. Representative epileptiform activity in mice electroporated with *RHEB*^{CA} . A. Interictal spikes not present in mice electroporated with control tdTomato. **B.** Examples of polyspikes. **C.** Examples of generalised seizures with spectrograms, associated with tonic-clonic convulsions.

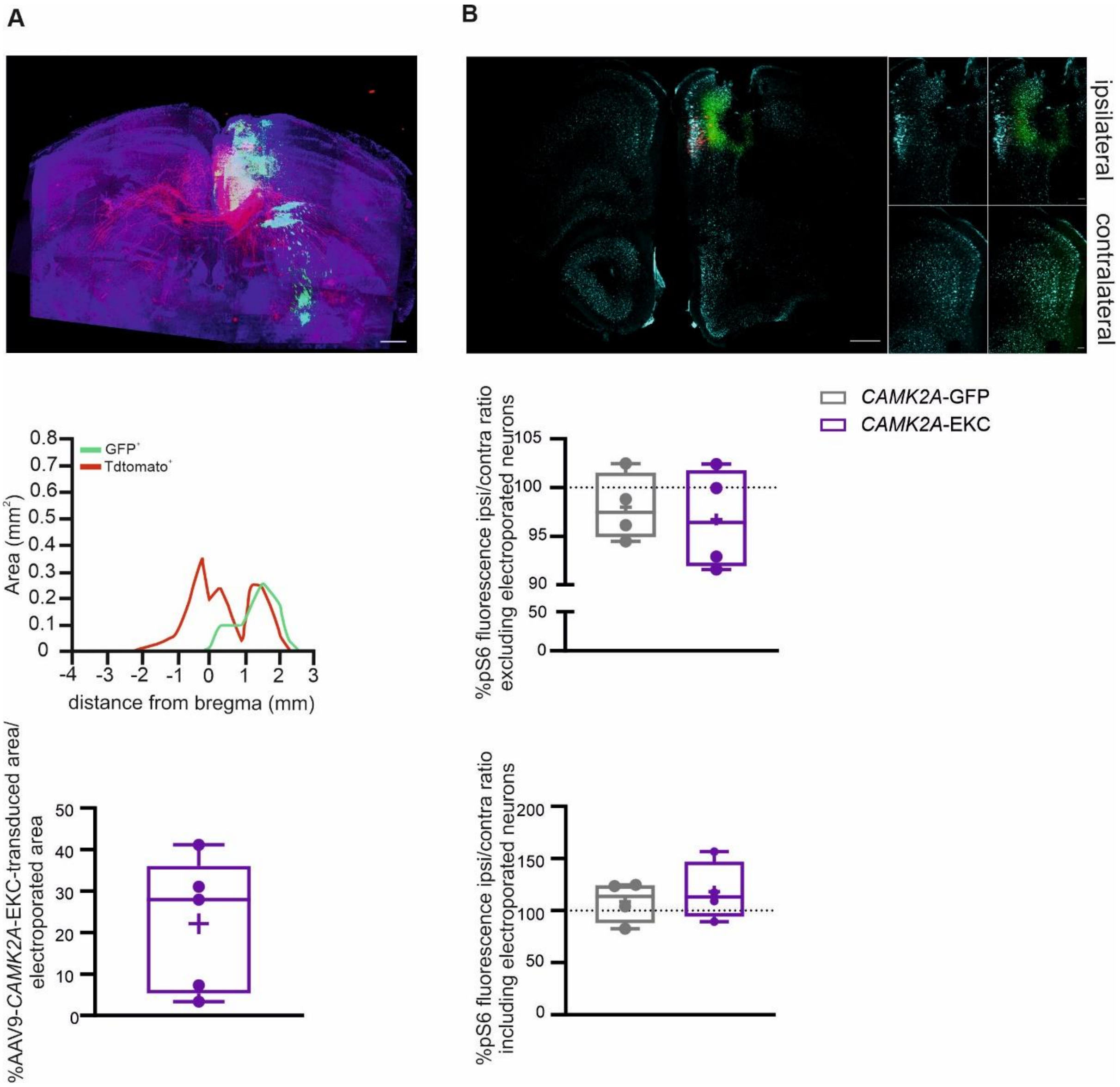

Fig. S3. ***CAMK2A*-EKC therapy spreads in the dysplastic region and does not reduce mTORC1 activity A.** 3D reconstruction of the spread of the dysplastic area (tdTomato, red fluorescence) and its overlap with expression of the GFP reporter included in the *CAMK2A*-EKC therapy, scale bar=500µm. (*middle*) Line graph displaying the anatomical overlap of the transduced (GFP^{+}) and electroporated (tdTomato^{+}) areas. (*bottom*) Box plot showing the percentage of electroporated area (tdTomato) overlapping with *CAMK2A*-EKC transduced area (GFP+) (n=5 *CAMK2A*-EKC-GFP mice). **B.** Immunofluorescence pictures of prefrontal cortical slice presenting a cortical dysplastic area (left panel: Two frontal hemispheres labelled with GFP (EKC) and pS6 antibody (cyan), scale bar=500µm; middle panels: Zoom in the ipsilateral hemisphere (top) and contralateral hemisphere (bottom) showing the pS6 expression (cyan); right panels: ipsilateral hemisphere (top) and contralateral hemisphere (bottom), scale bar=100µm . (*right*) Box plots displaying the % of pS6 fluorescence between the two hemispheres (ipsi = electroporated hemisphere) in animals from both groups (grey = *CAMK2A*-GFP, purple = *CAMK2A*-EKC, n=4 mice per group). Data are plotted as box and whiskers, representing interquartile range (box), median (horizontal line), mean (+ symbol) and maximum and minimum (whiskers), together with all the points.

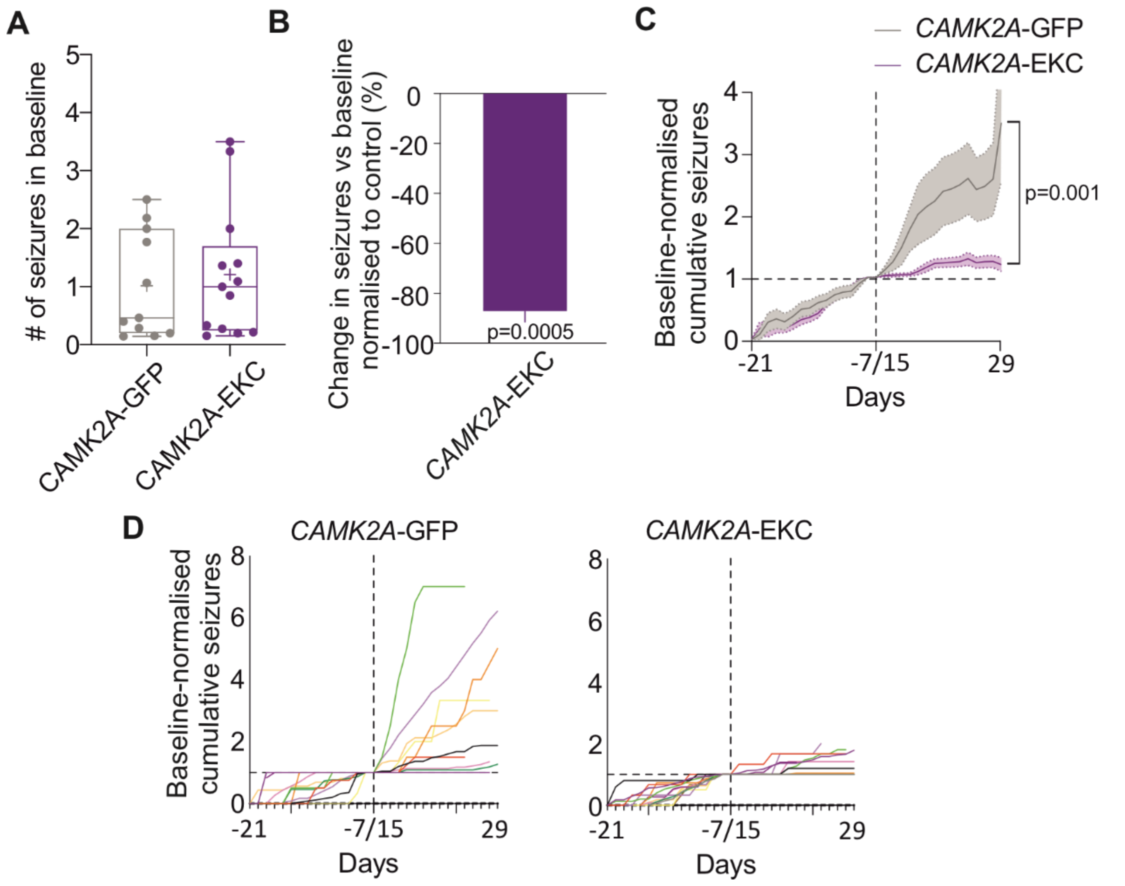

Fig.S4. *CAMK2A*-EKC and *CAMK2A*-GFP treated animals had similar baseline seizure frequencies. A. Box plot displaying the average seizure frequency recorded prior to AAV injection with either *CAMK2A*-EKC (n=13 mice) or *CAMK2A*-GFP (n=11 mice) (p=0.65, unpaired t-test). Data are plotted as box and whiskers, representing interquartile range (box), median (horizontal line), mean (+ symbol) and maximum and minimum (whiskers), together with all the points.
B. Graph displaying the change in number of seizures in animals treated with *CAMK2A*-EKC normalised to the average change in *CAMK2A*-GFP treated animals. **C.** Cumulative seizures normalised to baseline (*CAMK2A*-GFP n=11 mice, *CAMK2A*-EKC n=13 mice, Mixed effects analysis followed by Sidak's multiple comparison test). **D.** Cumulative seizures normalised to baseline for each animal; *left*: *CAMK2A*-GFP, *right*: *CAMK2A*-EKC

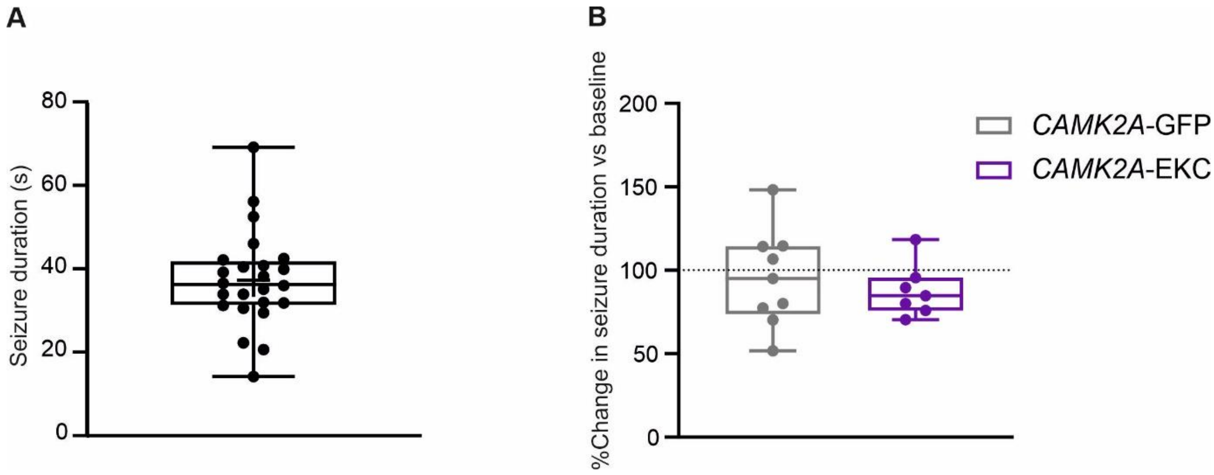

Fig. S5. *CAMK2A*-EKC therapy does not change seizure duration A. Box plot displaying the average durations of seizures recorded prior to AAV injection (n=24 mice). **B.** Box plot displaying the change in seizure duration normalised to baseline for *CAMK2A*-EKC (n=7 mice) and *CAMK2A*-GFP (n=9 mice) groups (p=0.5455, unpaired t-test). Data are plotted as box and whiskers, representing interquartile range (box), median (horizontal line), mean (+ symbol) and maximum and minimum (whiskers), together with all the points.

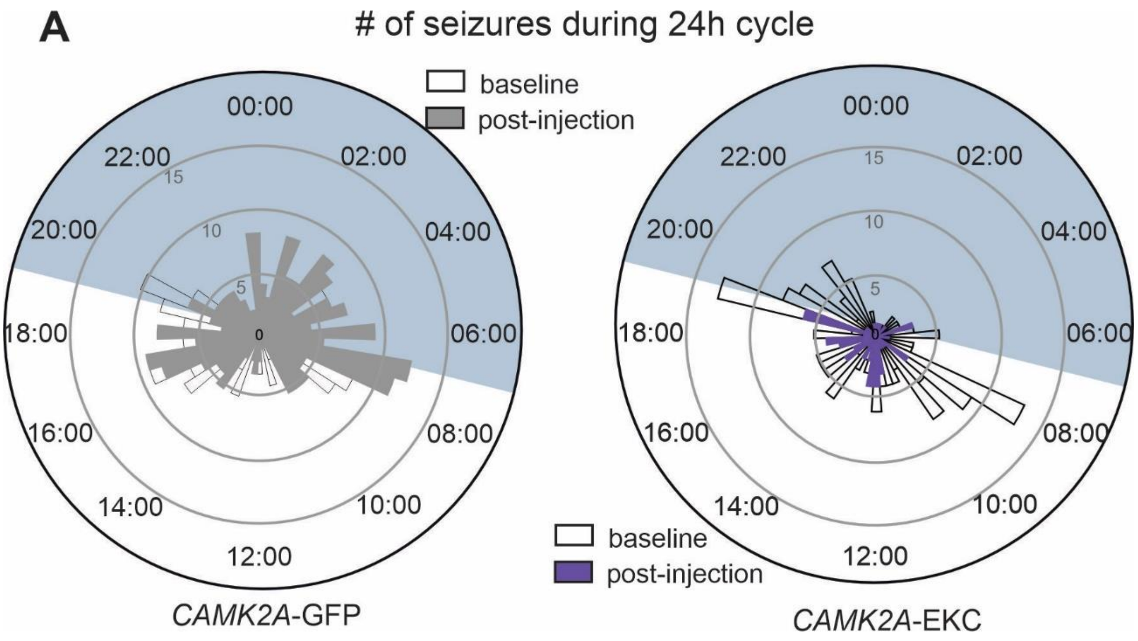

Fig. S6. *CAMK2A*-EKC therapy does not change the light-dark cycle pattern of seizure occurrence. Circular graph displaying the number of seizures before (empty rectangles) and after the therapy (filled rectangles) over 24h cycles for animals injected with either *CAMK2A*-GFP (n=11 mice) or *CAMK2A*-EKC (n=13 mice).

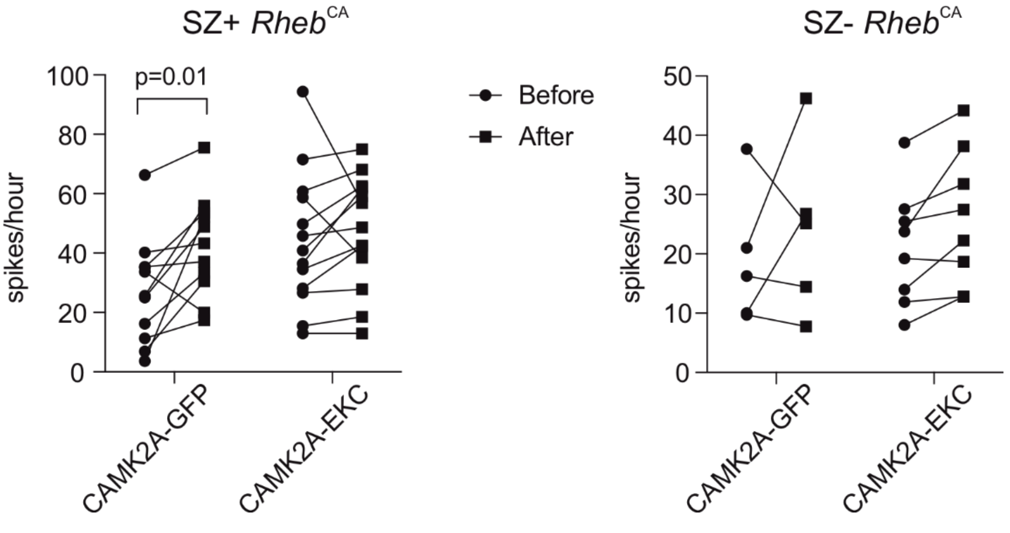

Fig.S7. *CAMK2A*-EKC therapy prevented an increase in interictal spike frequency in *RHEB*^{CA} animals with seizures. Slope graphs displaying spikes per hour in SZ+ *RHEB*^{CA} animals before and after treatment (*CAMK2A*-GFP n =11 mice, *CAMK2A*-EKC n =13 mice, 2-way ANOVA followed by Bonferroni multiple comparison test) and in SZ- *RHEB*^{CA} animals (*CAMK2A*-GFP n =5 mice, *CAMK2A*-EKC, n =8 mice).
